# You’re It—You’ve Got to Save Someone: Immediate Responders, Not Bystanders

**DOI:** 10.3389/fpubh.2019.00361

**Published:** 2019-12-05

**Authors:** Isaac Ashkenazi, Richard C. Hunt

**Affiliations:** ^1^Faculty of Health Sciences, Ben-Gurion University of the Negev, Be'er Sheva, Israel; ^2^United States Department of Health and Human Services, Washington, DC, United States

**Keywords:** bystanders, immediate responders, mass casualty incidents, emergency, response, disasters, terror, active shooter

## Abstract

Recurring disasters and life-threatening emergencies mandate that communities across the world be adequately prepared to prevent, respond, and recover from these events. Experiences throughout the world with mass casualty incidents and other disasters have increasingly highlighted the vital role that “active bystanders”—persons at the scene of an event who step forward to help—can play in preventing, containing, reporting, saving lives, decreasing morbidity, and increasing resilience. This paper seeks to emphasize the importance of the public in response to emergencies. No longer should we use the passive word “bystanders.” Rather immediate responders fill a critical silent gap before trained professionals arrive. In support of immediate responders this paper will identify the barriers to bystander action, and provide next steps to increase the number of individuals who take action at times of emergency. Immediate responders can and do play a valuable and unique role in reducing mortality, morbidity, and suffering from emergency events. While some cultures and countries have a long history of engaging the public as critical in an emergency response, others do not. The challenge is how best to increase the number of individuals who are motivated, prepared and ready to respond appropriately when they find themselves at the scene of an active shooter, bombing, hurricane, earthquake, tornado, fire, vehicle crash, or other life-threatening emergency.

## Introduction

Consider the following scenario: A terrorist attack injures many hundreds of people in <5 min. The numbers of patients far exceeds the numbers of trained responders. Life threatening bleeding will kill many before trained responders can arrive. This scenario that has already occurred and it remains a future possibility. How should those responsible for protecting people from harm prepare?

## Emergency Response Gap Filled by the Public

Emergency agencies across the world should carefully consider the role of the public during mass casualty incidents (MCI). In daily occurring emergencies resulting in a limited number of victims, emergency medical system (EMS), police and fire services bear the overall responsibility and have sufficient resources to manage the event and treat the wounded. In such routine events, the principal role for the public is to alert the appropriate emergency organizations, not direct or take action.

### The Silent Response Gap

There is a silent response gap—the time between the occurrence of the emergency incident and the arrival of trained responders, or in other words, the time between when people are injured, and the time that they receive treatment by first responders. During this critical period those injured can deteriorate clinically and can die even before responders arrive. This gap has not received attention by those leading emergency responses. This gap is composed of three sequential phases:

1. **No response**: scattered victims with multiple injuries that are not assisted by anyone except those who are administering self-aid.
2. **Immediate response**: active bystanders that step forward as immediate responders to support the victims.
3. **Intermediate response**: a combined response by immediate responders (active bystanders) and trained first responders. This phase exists particularly in countries in which the activism of immediate responders is acknowledged and embraced such as Israel, Spain, and India.

There always will be a potentially life-threatening time delay between the occurrence of an incident resulting in mass casualties and the arrival of official trained responders. Even in planned mass gathering events, e.g., concerts, festivals, marathons, etc., where emergency personnel are prepositioned, there are delays from when an incident occurs until a patient is treated. The delay can result from impeded access to patients and/or from the numbers of patients exceeding the capacity of the pre-positioned on scene personnel.

An example of delay in accessing patients was identified in the Kerslake Report, an independent review into the preparedness for, and emergency response to, the Manchester Arena attack on 22nd May 2017. The report identified that “The Greater Manchester Fire and Rescue Service (GMFRS) did not arrive at the scene and therefore played no meaningful role in the response to the attack for nearly two hours. This compares with an average response time for the Service of less than six minutes. The effect of this was that a valuable resource was not available to assist on the scene….”

In MCIs with very large numbers of victims, the public is best positioned to provide immediate support because of their proximity to the injured. The distance and obstacles in the way of official trained emergency responders preclude their ability to access victims rapidly and to treat all victims simultaneously. Experience has shown that the public can and does help by offering both physical and emotional support to those in need.

## Definitions

Those members of the public at the scene of daily occurring emergencies and mass casualty incidents traditionally have been called “bystanders.” The Oxford Living Dictionaries definition of bystander is “a person who is present at an event or incident but does not take part” (1). Synonyms for bystander include passerby, witness, eyewitness, spectator, looker-on, and eye-witness.

Many countries provide phone numbers for their citizens to summon emergency aid. Bystanders in many countries identify the need for emergency aid and call for help. These are important steps in transitioning from “standing by” to taking action. As an example from one country, for decades professional trained emergency responders in the United States have encouraged the public to play a critical role in accessing emergency response by calling 911.

The critical role that “bystanders” play in saving lives is exemplified by their role in response to sudden cardiac death. For cardiac arrest delays in cardiopulmonary resuscitation (CPR) and defibrillation decrease the chance for survival. There have been substantial efforts in the United States and other countries to train the public in CPR and in automated external defibrillation (AED), and to encourage them to act in response to cardiac arrest prior to the arrival of official trained responders. Until recently in the United States, with the exception of CPR and using AEDs for cardiac arrest, the public traditionally has stood by and stayed out of the way of trained responders.

Sudden cardiac death is a single patient event in contrast to large scale overwhelming no-notice terrorist attacks resulting in many casualties. Yet the challenges and successes in engaging the public to respond to cardiac arrest informs us how we can better engage the public to respond to large numbers of casualties.

“Bystander” generally has been an appropriate term; the public has been standing by. However, in recent years the public has shown an increasing propensity to respond immediately. This cultural shift in the United States has not yet been fully realized. In the case of a MCI, immediate responders can be defined as anyone who is present at the scene of an emergency event. “Bystanders” can be either active (helpers) or passive (not helpers). Active “bystanders” are the immediate responders—those who step forward to help. Ideally, everyone should know basic lifesaving skills. Yet those on the scene of a MCI are not likely to have specialized knowledge or equipment for lifesaving However, as a result of being at the scene during the MCI, they are the only individuals in a position to provide immediate potentially lifesaving assistance.

They are a group united by time, space, and the common experience of being present together at a horrific incident. They are joined together in their most desperate moments of life. Their common cause is survival.

The Boston Marathon bombing and the Las Vegas 1 October active shooter incident in the United States highlights the beginning of a cultural change in how the public in one country responds during mass casualty events. Boston has always had robust medical preparedness plans for the Boston Marathon, including a well-staffed medical tent for the marathon runners, and Boston has one of the highest concentration of trauma centers in the United States. Yet, even with those resources, the numbers of casualties required bystanders to help save lives. Similarly, Las Vegas has extensive prehospital and hospital preparedness and response capacity and capabilities, especially since it is an international tourist destination Yet the number of casualties on 1 October 2016 exceeded the on-scene capacity to care for them all fast enough. In Boston and in Las Vegas “bystanders” did not stand by. Many stayed or went into harm's way. The person next to them would die if they didn't act to stop life threatening bleeding.

Different cultures have different approaches to the public's role in a mass casualty incident. Israel's flow of casualty care, the descriptions by medical response leaders of the public responses to civilian bombings in different countries during the Tale of Cities conferences (2), and the evolution of bystanders in the United States inform our understanding of the critical role of the public in MCIs.

The paradigm about bystanders needs to change. The word “bystanders” needs to be replaced by the term “immediate responders.” Thus, the term “bystanders” has been changed to “immediate responders” throughout the remainder of this paper.

## Flow of Casualty Care (Israel)

Israel has a culture that has embraced the entire public as one of their pillars of casualty care (3).

There are five pillars of casualty care: Immediate Responders; Trained Official Emergency Responders (official emergency entities); Distribution System; Hospital Care and Post Hospital Care.

To protect the bystanders and to encourage helping behavior, in 1998, Israel passed the Good Samaritan law which shields the active bystander from civil liability, and mandates that a bystander MUST assist an individual who he sees is in serious danger to his/her life due to a sudden event. Furthermore, this law states that the bystander is entitled to compensation for the costs, damages and health problems that are associated to the rescue (4).

## The Israeli Operational Timetable for Responding to MCI

The timeline was developed by this paper's principal author during his tenure as Surgeon General of the Home Front Command. The purpose of the timeline is to save lives, decrease morbidity and increase the resilience of the public. The message to the public is: if you are a victim, we will do everything in our power to take care of you. You will receive the best care available, immediately! The timeline is based on five milestones (“deadlines”):

**Within 5 min**: The commander should be on scene. At the onset of the event, the commander does not have to be an official responder that approached the area. Any immediate responder willing to lead may take control. If you want it, it's yours—get in there and start directing the lifesaving effort.

**Within 20-min**: all victims initially are treated and cleared from the scene. Everyone is authorized to help. Everyone is EXPECTED to help. Moving rubble off of victims, applying direct pressure to stop the bleeding and delivering patients to the appropriate medical facility. The “bystanders” are the immediate responders! Waiting for ambulances or until SWAT declares the area “safe” will not help as many victims as will getting victims to the trauma center immediately.

Many times, the first ambulance arrives on scene with only a driver. An immediate responder may care for the victim in the ambulance while the driver drives to the hospital.

**Within 60-min**: all victims are being treated in hospitals. Along with that, the press is invited to interview any patient that who is willing, and the doctors involved give regular updates to the press with an emphasis on how well the survivors are treated at the hospitals.

**Within 180-min:** scene is completely cleared of signs of destruction, flesh, blood, evidence, and roads are open. A couple of extra hours may be requested under special circumstances, but investigators can get all the evidence they need in that time. Police do not own the scene—public property belongs to the public. Emergency agencies should be quick, and gone.

**Within 2–4 days:** the area should be reconstructed. The scene is back to normal. Allowing the crime scene to stand longer than that turns it into a shrine for terrorism. Keeping it in the news cycle and continuing to project the graphic images across the nation reduces public resiliency and further supports the terrorists' message. On the other hand, the image of the scene returning to normal and people going about their lives demonstrates resiliency! That teaches a lesson to the enemy: You will never win. You will never defeat us. Your actions will not alter our daily lives!

## Bystanders in Israel are the First Preventers

The public has a critical role in the immediate response after an incident occurs. However, Israel has demonstrated the public also has a role in preventing the occurrence of attacks. In the Israel experience with suicide attacks, there was substantial risk to those preventing the attack with a marked decrease in casualties.

Out of 103 suicide attacks from 2000 to 2003, 40 experienced interventions by bystanders. Tragically, immediate responder interventions to prevent an attack nearly always triggered an attack, killing the Good Samaritan. However, interventions by immediate responders reduced casualties by more than 70%. Immediate responders prevented attackers from selecting optimal locations (5).

Bystanders are the immediate responders. They are the most important actor in crisis. They are the first to prevent, report, respond, and command. They are the first to save lives, decrease morbidity, and elevate resilience.

## Tale of Our Cities

Realizing that the world's experiences with MCIs were crucial to improving response, CDC's Division of Injury Response, with support from the HHS Office of the Assistant Secretary for Preparedness and Response and in collaboration with Harvard University's National Preparedness Leadership Initiative and multiple injury care stakeholders, supported the Tale of Our Cities Initiative (TOCI) during 2008–2011. Through a multi-city series of workshops in Boston, New York City, Seattle, Washington DC, San Diego, Houston, Chicago and additional cities, the international response leadership from Israel, Madrid, Pakistan, London, Mumbai, and Delhi, shared their experience in preparedness and response to such MCI's (2).

What surprised all attending the TOCI workshops was the similarity in experiences across countries. A common theme that emerged among the international speakers was the major role of the public during responses to civilian terrorist bombings. Given the disparities among international health systems and cultures, the uniform acknowledgment by each international expert that the response by the public was critical in saving lives after large scale terrorist bombings was unexpected.

The phrase “a picture is worth a thousand words” powerfully reinforces our need to reevaluate our understanding of “bystanders.” Photographs and videos from many different mass casualty events are so very telling: many casualties lying on the ground, and many people sitting, kneeling or standing at the casualties' sides. Some appear to be providing care or comfort; some appear to possibly be injured as well. The lines blur when trying to distinguish who's a victim and who's an active immediate responder providing care. It may be that, in that moment, the individuals themselves weren't sure if they were a victim or an active immediate responder.

Videos from MCIs show people running away from incidents that could cause them harm, but within seconds many return to save the victims during probably the most dramatic life-threatening circumstance of their lives. For example, a sudden storm caused a stage collapse at the Indiana State Fair in 2011. People are seen running away from the stage as it collapses and then returning to help. In the immediate aftermath of a large-scale incident, there are far more active immediate responders than trained official responders providing care or comfort to casualties (6–10).

Personal experience (Ashkenazi), photos and videos tell us that in disasters resulting in hundreds and thousands of casualties there simply aren't enough professional responders to save lives.

There always will be a delay (the silent gap) before an official emergency response. In mass casualty events with dozens, hundreds or thousands of victims, the unharmed public is in the best position to provide immediate assistance because (1) their proximity to the injured, and (2) obstacles in the way of official emergency responders that preclude their ability to access victims rapidly and treat all victims simultaneously. Experience has shown that the public CAN AND DOES help by offering both physical and emotional support to those in need.

The paradigm about bystanders needs to shift. Changing our terminology from “bystanders” to “immediate responders” supports what has been observed in response to multiple disasters across the world, may increase professional responders' engagement of immediate responders, and recognizes the critical role immediate responders have in saving lives in the silent gap.

## Facts and Principles that Should Guide Our Thinking About Immediate Responders

1. There never will be enough professionals to save lives in large mass casualty events. Professionals cannot ever be there fast enough unless they are on the scene when the event occurs.
2. The public is always at the scene at the moment the event occurs. They are there at the right time, at the right place.
3. Trained official emergency responders in the United States traditionally have not been accustomed to embracing and empowering bystanders at the scene.
4. The public will always outnumber professional rescuers. In the United States.
  - a. First responders: Firefighters about 1.2 million; police over 900,000 sworn police officers, EMS credentialed professionals about 826,000 (11– 13).
  - b. Immediate responders: Over 320 million potential active bystanders.

While the training and experience of the official emergency responders is greater, the number and availability of the public is dominant.

## Obstacles Decreasing the Propensity for the Public to Take Action

Immediate responders may be reluctant to help because of many fears including liability, self-injury, HIV, hepatitis, lack of knowledge, and concern about hurting rather than helping. In addition, emergency professionals in some cultures and countries frequently discourage immediate responders from helping. Plans, training and policies for preparedness and response generally have not included the public as partners that can help save lives.

Research is needed to understand how immediate responders and trained responders can best work together in a response. That research should take into consideration the fact that immediate responders will be the only responders who can save the lives when trained responders can't arrive soon enough or the numbers of injured exceed the number of responders.

## “If You See Something, Say Something” Campaign

The engagement of the public as immediate responders in the primary prevention of terrorist attacks with the US Department of Homeland Security's “If You See Something, Say Something” campaign is an encouraging effort to include the public in preparedness and response. However, the campaign can go further than the request to report.

We propose taking a step forward: acknowledge the fact that immediate responders are involved and will be involved in any disaster response- not as victims, but as immediate responders. “If You See Something, Say Something, *Do Something*” can move us beyond reporting to taking action.

## Flee From or Run To

People are conflicted about whether or not they should flee from or run to the aid of others in life threatening circumstances. The United States has made some progress toward enabling the public to be immediate responders to MCIs. Even before September 11th, the American Red Cross, the Boy Scouts, and other organizations were involved in first aid education. Since September 11th there have been significant efforts, such as those by the Community Emergency Response Teams, to train the public in first aid CPR and more recently the Stop the Bleed. Yet, those initiatives require a member of the public to be motivated to learn, and none of the initiatives have yet reached the entire public. In many major cities in the US, the public is disconnected from emergency preparedness and response. Instead of increasing the public's propensity to prepare and respond, they may be perceived by emergency response leaders as victims rather than helpers.

Bystander CPR and Automatic External Defibrillator education programs have been major initiatives for many years by the American Heart Association, and are responsible for saving many lives. However, CPR and automatic defibrillators are not the skills that will save lives in large-scale disasters resulting in life-threatening injuries, such as earthquakes, tsunamis, multiple simultaneous terrorist bombings, or active shooters. For MCI's resulting in injuries the public's ability to control bleeding can save lives.

Having the entire general population of countries become “immediate responders” is a major challenge. Whether a layperson has an interest in being trained is certainly influenced by their self-perceived risk of exposure to the vector causing life threatening illness or injury, availability of training, and whether or not he/or she has been encouraged to get trained. Those factors will vary considerably by country.

People who have the cognitive and physical skills required for the intervention can learn simple lifesaving knowledge and skills, such as CPR, automatic external defibrillation, and bleeding control. The American Heart Association indicates that studies have shown that children as young as 9 years old can learn and retain CPR skills. There is insufficient research to determine the minimum age for which a child has the cognitive and physical skills needed to perform lifesaving bleeding control.

A reasonable beginning for engaging immediate responder is to focus on populations that would have a higher risk of being present at mass casualty event. For example, training those who work in locations where prior mass casualty events, such as those that have occurred in public spaces such as transportation hubs, schools, stadiums, and concert venues, can be an important initial step leading to an expanded initiative.

## Recommendations

Take this article a step forward and acknowledge the fact that in many countries and cultures the public has been involved and will be involved in any disaster response- not as victims, but as immediate responders. We propose that leaders of emergency response across the globe support the public as immediate responders-partners in saving lives and building resilience.

- The word “bystanders” should be replaced by “immediate responders.”
- Emergency preparedness and response leaders must develop a new understanding of the public's behavior, motivation, capabilities, and expectations.
- “Immediate responders” should be incorporated into emergency planning and response. Emergency planners should consider carefully ways to incorporate the public into their plans and protocols.
- Policy makers should consider how to best empower, train and equip their population to save lives.
- National Good Samaritan laws, like that of Israel's, support the reality that disasters cross state boundaries and should be supported.
- Shift the professional paradigm from discouraging “bystanders” from helping to engaging them as immediate responders into a collective mission of saving lives that crosses boundaries.
- Shift the paradigm of the public being fearful of playing an active role in saving lives to being engaged and empowered to be life savers.

For many countries implementing the above recommendations will require evolution in culture. Choosing a simple near term success can accelerate that evolution. As an example, Stop the Bleed was chosen in the United States because control of life threatening bleeding required simple steps that everyone can learn, and could save lives from daily occurring emergencies and disasters. Choosing receptive audiences early on gains a foothold from which to expand implementation and then add other lifesaving skills. Since the 2015 launch of Stop the Bleed over one million people have been trained and there are programs in over 100 countries. Another example accelerating cultural change is life-saving skill training in schools and/or the military, making these skills part of the cultural norm.

Having every citizen know how to save lives in a disaster likely will save lives in those emergencies that occur every day, and it will build personal and public resilience.

## Limitations

While there may be other countries and/or cities that have experienced or supported “immediate responders” in their preparedness and response efforts, this paper is limited to the description of those of Israel, the United States, and of the Tale of Cities Initiative: Israel, Madrid, Pakistan, London, Mumbai, and Delhi.

## Implications—An Empowered Public

Under the threat of any kind of violence or emergency a society can help protect itself by supporting immediate responders. Societies, cultures, and especially trained responders should trust immediate responders; they are an asset and not an obstacle. Those responsible for protecting people from harm should (1) prepare immediate responders to prevent, contain, and report threats and respond appropriately, (2) share with the public information, responsibility, and expectations, (3) train immediate responders to respond appropriately to save lives and start training at schools, and (4) acknowledge immediate responders for helping behavior.

## Author Contributions

All authors listed have made a substantial, direct and intellectual contribution to the work, and approved it for publication.

### Conflict of Interest

The authors declare that the research was conducted in the absence of any commercial or financial relationships that could be construed as a potential conflict of interest.
